# Feasibility of an ADAPTive intervention to improve food security and Maternal-Child Health (ADAPT-MCH): Protocol for a pilot sequential multiple assignment randomized trial

**DOI:** 10.1016/j.cct.2025.108086

**Published:** 2025-09-18

**Authors:** Deepak Palakshappa, Rebecca J. Stone, Brenda Ramirez, Sarah E. White, Joseph Rigdon, Richa Bundy, Sally G. Eagleton, Nicole Caudill, Heather Martin, Mayte Grundseth, Scott Best, Morgana Mongraw-Chaffin, Kristina H. Lewis, Kimberly Montez

**Affiliations:** a Department of Internal Medicine, Wake Forest University School of Medicine, Winston-Salem, NC, USA; b Department of Pediatrics, Wake Forest University School of Medicine, Winston-Salem, NC, USA; c Department of Epidemiology and Prevention, Wake Forest University School of Medicine, Winston-Salem, NC, USA; d Department of Obstetrics and Gynecology, Wake Forest University School of Medicine, Winston-Salem, NC, USA; e Department of Biostatistics and Data Science, Wake Forest University School of Medicine, Winston-Salem, NC, USA; f Second Harvest Food Bank of Northwest NC, Winston-Salem, NC, USA; g Forsyth County Department of Public Health, Winston-Salem, NC, USA; h Help Our People Eat (H.O.P.E), Winston-Salem, NC, USA; i Department of Population Health Research, MedStar Health Research Institute, Columbia, MD, USA; j Department of Social Sciences and Health Policy, Wake Forest University School of Medicine, Winston-Salem, NC, USA

**Keywords:** Food insecurity, Pregnancy, WIC, Produce prescriptions, Medically-tailored meals, Feasibility studies

## Abstract

**Background:**

Food insecurity affects up to 30 % of pregnancies and is associated with worse maternal and infant health. Healthcare systems are implementing interventions to assist patients with food insecurity, but rather than providing a single intervention, adaptively providing interventions could be a more effective strategy. The objective of this study is to determine the feasibility of adaptively providing interventions to assist pregnant patients who report being food-insecure.

**Methods/design:**

We will conduct a pilot sequential multiple assignment randomized trial at obstetrics clinics from one health system. Adults (*N* = 60) who are pregnant and food-insecure will be randomized at their initial prenatal visit to one of two first-stage interventions for 3 months: 1) electronic health record (EHR) referral to WIC or 2) EHR-referral to WIC + care navigation. Participants who do not have ≥2-point improvement in food insecurity after 3 months will be re-randomized to one of two second-stage interventions for an additional 3 months: weekly delivery of 1) produce or 2) medically-tailored meals. In Aim 1, we will determine the feasibility of recruitment, and in Aim 2, we will evaluate the feasibility of re-randomization, retention, and data collection. In Aim 3, we will advance our understanding of how, why, and under what circumstances participants achieved improvements through semi-structured interviews.

**Conclusions:**

This will be the first study to test an adaptive intervention to assist pregnant patients with food insecurity and will inform a future fully-powered trial. Given the growing interest among health systems, an efficacious, adaptive food insecurity intervention could be broadly disseminated.

**Trial registration:**

The study was registered with ClinicalTrials.gov (NCT06942598) on April 23, 2025.

## 1. Background

The U.S. is facing a maternal and infant health crisis with approximately 700 maternal deaths and 50,000 life-threatening pregnancy events occurring each year [1–3]. Maternal deaths in the U.S. are now less likely to be due to direct complications of childbirth and are increasingly due to endocrine (e.g., gestational diabetes mellitus) or cardiovascular (e.g., pre-eclampsia) conditions directly related to nutrition-related chronic diseases [4,5]. One significant contributor to maternal morbidity and mortality is food insecurity (FI), or the lack of consistent access to the food needed for a healthy life [6,7]. In 2023, 13.5 % of U.S. households were food insecure, and up to 30 % of pregnancies are impacted by FI [8,9]. FI has been associated with inadequate or excessive gestational weight gain, gestational diabetes mellitus, and pregnancy-induced hypertension as well as increased risk of preterm birth and infants being born low birth weight [9–13].

To mitigate the impact of FI on health, national healthcare organizations, such as The American College of Obstetricians and Gynecologists, have recommended that health systems address FI as a routine part of clinical care [14–16]. The integration of interventions to address FI in clinical care settings has been termed “Food is Medicine” [17–19]. Three Food is Medicine interventions that are being used include: 1) referring patients to government benefits intended to support nutrition (e.g., the Special Supplemental Nutrition Program for Women, Infants, and Children (WIC)) or directly providing food through the use of 2) produce prescriptions (Produce Rx) and 3) medically-tailored meals (MTM) [19–21].

Despite the growing use of Food is Medicine interventions, research on the impact of healthcare system-based interventions to reduce FI in pregnancy remains limited [22,23]. Additionally, studies have found variability in the success of any one FI intervention [24,25]. For example, studies referring patients to government programs found only 30–40 % of patients received new benefits [26,27]. Although, Produce Rx and MTM may be more effective than referrals alone, they are costlier and more resource intensive, which could limit broad dissemination and scalability [24,25].

Rather than offer a single intervention, adaptively providing Food is Medicine interventions could provide a more equitable and efficient approach to assist pregnant patients. Adaptive, or stepped-cared, interventions provide a lower resource intensive intervention first, and then a higher resource intensive intervention to those more in need or who do not respond to the initial intervention [28–30]. However, there have been no adaptive health-system integrated interventions tested to assist pregnant patients with FI. To address this gap in the literature, we will conduct a pilot sequential multiple assignment randomized trial (SMART) to test the feasibility of adaptively providing Food is Medicine interventions to pregnant patients.

## 2. Methods

### 2.1. Ethics statement

The Wake Forest University School of Medicine Institutional Review Board (IRB) has reviewed and approved all the procedures outlined in this protocol. The study was prospectively registered with ClinicalTrials.gov (NCT06942598) prior to recruitment of the first participant. We have also included an independent safety officer who will review participant data to assess for any adverse events.

### 2.2. Study setting

This study will include patients from Atrium Health Wake Forest Baptist (AHWFB). AHWFB is a clinically-integrated, academic medical center comprised of the Atrium Health Wake Forest Baptist Medical Center, the Wake Forest University School of Medicine, and outlying affiliated hospitals and physician practices. AHWFB serves patients at over 350 care locations. We will conduct this study at obstetrics clinics affiliated with AHWFB.

### 2.3. Overall study design

Guided by the impact of FI on health and Food is Medicine conceptual frameworks [17,31–33], the objectives of the “Feasibility of an ADAPTive intervention to improve food security and Maternal-Child Health (ADAPT-MCH)” are to: 1) determine the feasibility of recruitment and retention and 2) generate preliminary estimates of the potential effectiveness of the interventions in anticipation of a large, definitive SMART to improve FI. A fully-powered SMART is needed to answer two questions: 1) as WIC is an evidence-based program shown to reduce FI and improve pregnancy outcomes, is a direct electronic health record (EHR) referral to WIC enough to reduce FI or do pregnant patients not already enrolled in WIC need a referral plus assistance from a care navigator? 2) for people who do not have an improvement in FI from the initial intervention, should healthcare settings provide Produce Rx or MTM? This pilot study is necessary to determine the feasibility of recruitment, re-randomization, retention, data collection, and adaptively providing FI interventions [34]. Data from this study will provide information to refine the study protocol and estimate measures of variance to assist in sample size calculations to inform a future fully-powered SMART.

**Fig. 1.**
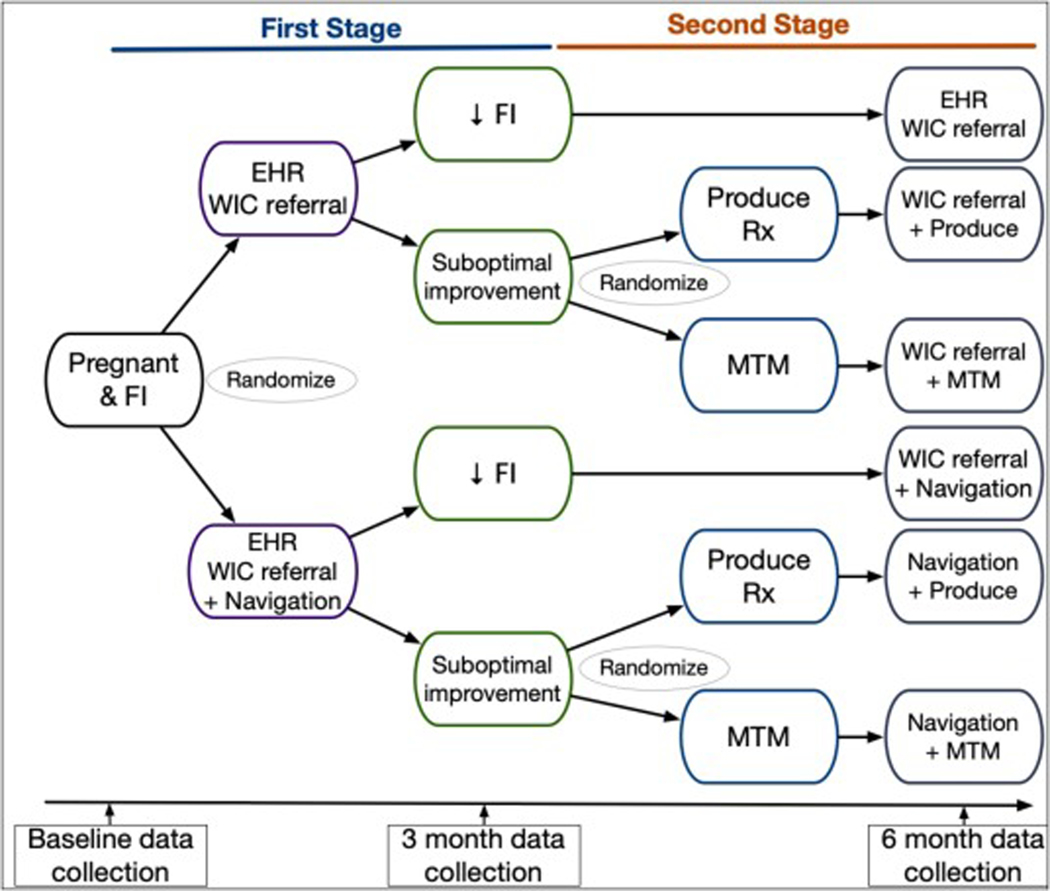
Overall study design. We will conduct a single site pilot sequential multiple assignment randomized trial (SMART). Patients (N = 60) who are pregnant and food insecure will be randomized to receive one of 2 interventions in Stage 1:1) EHR referral to WIG or 2) EHR WIC referral + care navigation. We will follow up all participants at 6 months Participants who do not have ≥2 point decreased in food insecurity at 3 months (suboptimal improvement) will be re-randomized to one of 2 interventions in Stage 2: 1) Produce Rx or 2) MTM. We will follow up all participants at 6 months. EHR-electronic health record; MTM-medically tailored meals; Produce Rx-produce prescriptions; WIC-Special Supplemental Nutrition Assistance Program for Women, Infants, and Children.

The proposed pilot study is a single site two-stage SMART (Fig. 1). We will enroll 60 patients who are pregnant and food-insecure from obstetrics clinics affiliated with AHWFB. Using a computer-generated randomization table to ensure allocation concealment, eligible consenting participants will be equally randomized using permuted blocks of size 2,4, or 6 (1:1) to 1 of 2 first-stage interventions: 1) EHR referral to WIC or 2) EHR referral to WIC + care navigation. We will re-assess all participants at 3 months. Participants who do not have ≥2-point decrease in FI based on the 10-item adult USDA Food Security Survey Module (FSSM) (suboptimal improvement, i.e., non-responders) will be equally re-randomized using permuted blocks of size 2, 4, or 6 to 1 of 2 second-stage interventions: 1) Produce Rx or 2) MTM [7]. The USDA FSSM with a 30-day look back period measures food security over the prior 30 days. We will use the standardized scoring provided by the USDA, which produces a raw score from 0 to 10 with higher scores indicating worse FI. Due to the nature of the intervention, neither study staff nor participants are blinded to their assigned group.

In Aim 1, we will determine the feasibility of recruitment and which first-stage intervention is potentially more effective in reducing FI. In Aim 2, we will evaluate the feasibility of re-randomization, data collection, and retention and compare preliminary estimates of change in FI of providing Produce Rx versus MTM among participants who did not respond to the first-stage intervention. In Aim 3, we will advance our understanding of how, why, and under what circumstances participants achieved improvements to the interventions. Participants will engage in the interventions for up to 6 months, and we will follow all participants to after they have delivered. A 6-month intervention period was selected in keeping with similar FI studies [22,23]. As secondary outcomes, we will evaluate maternal and infant outcomes (Table 2) including the number of participants who are diagnosed with gestational diabetes, have excess gestational weight gain, pre-eclampsia, infant birth weight, and pre/post-term delivery based on data extraction from the EHR. Based on the impact of FI on health conceptual framework, we will also assess changes in nutritional, psychological, and compensatory pathways from baseline to 6 months [31–33]. As an exploratory aim, participants will wear a continuous glucose monitor (CGM) at 2 time points: baseline (first 2 weeks) and at 6-month follow up.

### 2.4. Recruitment and consent

As part of routine care at the participating obstetrics clinics, any patient who believes they may be pregnant undergoes a pregnancy test and an ultrasound for confirmation of pregnancy followed by a nurse intake visit if a viable pregnancy is identified. The nurse intake visit is ideally performed at 8 weeks gestation. Patients then return 1–2 weeks later to meet the obstetrician or advance practice provider for an initial prenatal visit and begin prenatal care. During the initial 8-week nurse visit, all patients are screened for FI using the validated 2-item Hunger Vital Sign^™^ [35]. The measure asks: 1) “Within the past 12 months you were worried that your food would run out before you got money to buy more”, and 2) “Within the past 12 months the food you bought just didn’t last and you didn’t have money to get more.” Participants who respond “Often true” or “Sometimes true” to either question are considered food insecure, and responses to the items are captured as structured data elements in the electronic health record (EHR). Compared to the USDA FSSM (gold standard for assessing food security) [7], this 2-item measure has high sensitivity (97 %) and good specificity (83 %) and has been used in numerous prior studies in clinical settings [35–38]. Given this is a pilot study with a small sample size and to allow the interventions time to take effect, we will only include patients who are presenting in their first trimester (exclude patients presenting for the initial nurse visit in their 2nd or 3rd trimester). Using structured EHR data and a recurring weekly query, our research team will call all patients who are potentially eligible prior to their initial visit with the obstetrician/midwife to introduce them to the study and determine interest in participating. Potentially eligible and interested patients will be approached in the clinic at the time of their visit to assess eligibility, discuss study procedures, obtain consent, and collect baseline data. Patients will be eligible if they meet the prespecified inclusion and exclusion criteria (Table 1). After baseline data are obtained, participants will be randomized. We will continue this process until we reach our target sample size of 60 (see Power and Sample Size).

### 2.5. Interventions

As part of usual care, all participants in the study will receive routine prenatal care from their regular obstetrics team. Though FI often goes untreated in clinical care, not offering assistance for FI in a research setting poses unacceptably high risks to participants. We therefore propose a comparative effectiveness design, initially comparing HER-based WIC referral to WIC referral + care navigation. As both strategies are plausibly beneficial, but neither is known to be efficacious for improving FI or maternal-child health nor superior to the other, the principles of clinical equipoise are met.

#### 2.5.1. Stage 1 interventions

1. EHR WIC referral: Participants randomized to this intervention will be referred to the WIC program through an already developed electronic referral process [39,40]. The process securely sends a referral to WIC program staff via the EHR. To enable WIC offices to electronically receive referrals and communicate with healthcare teams, our EHR offers a community provider-facing, read-only version which WIC staff have been certified and trained to use under an existing memorandum of understanding between AHWFB and county health departments.
2. EHR WIC referral + care navigation: Participants will receive the same intervention as above. In addition, a bilingual patient care navigator will meet with the participant at enrollment to discuss any barriers to accessing WIC. The purpose of the visit is to build rapport and trust and to identify any social or structural barriers to enrolling in WIC. The navigator will also contact participants at 2 weeks to discuss any additional barriers reported and as necessary after the baseline visit. Specific counseling will be tailored based on individual’s needs (i.e., difficulty with paperwork). The navigator will also assess any additional community resources available to assist the participant (e.g., food pantries). All encounters between the navigator and participant will be documented and reviewed by the study team to assess fidelity.

#### 2.5.2. Suboptimal improvement

We will re-assess all participants at 3 months using the 10-item FSSM. Participants who do not have a ≥ 2-point decrease in FI, whether they enrolled in WIC or not, will be re-randomized to 1 of 2 second-stage interventions. We selected a decrease of 2 points and time horizon of 3 months based on prior studies, which have shown that receipt of government benefits (WIC, Supplemental Nutrition Assistance Program (SNAP)) results in approximately a 2-point decrease in FI over that time period [22,23,41]. Although studies have shown that interventions to address FI during pregnancy improve food security, prior studies have not quantified the amount of change in FI and differences in pregnancy outcomes. Previous studies have shown a 2-point decrease in FI is associated with clinically meaningful improvements in quality of life and mental health [42].

#### 2.5.3. Stage 2 interventions

1. Produce Rx: Participants randomized to this arm will receive $10 worth of fruits and vegetables each week from our community partner, Help Our People Eat (H.O.P.E.) of Winston Salem. H.O.P.E. is a non-profit that distributes nutritious meals and fruits and vegetables to neighborhoods throughout Forsyth County to help families with FI. H.O.P.E. also collaborates with local farmers to provide a Neighborhood Produce Market that provides low-cost produce. Through H.O.P.E’s prior work, $10 of produce provides enough fruits and vegetables for a family of 4 for 2–3 days. Weekly deliveries of produce will be made to a participant’s home for 3 months, include a mix of fruits and vegetables depending on the season, and provided by local farmers.
2. MTM: MTM will be provided by our community partner, Providence Kitchen, a program of Second Harvest Food Bank of Northwest NC. Participants will receive 10 medically-tailored refrigerated or frozen meals (5 lunches and 5 dinners) delivered to their home weekly for 3 months. All meals are planned by a registered dietician and tailored to meet the nutritional needs of pregnant women. Meals will require minimal preparation time, and will be packaged such that they can be re-heated by stove, oven, or microwave. Participants will be asked not to share the meals with family members. Consumption of meals and food sharing with other household members will be measured using food consumption diaries.

### 2.6. Outcomes

We will assess outcomes based on the impact of FI on health and RE-AIM conceptual frameworks [31–33,43–46]. We will focus on 3 parts of RE-AIM (Reach, Effectiveness, and Implementation). “Reach” refers to the proportion of the target population who participate, “Effectiveness” to the impact on the desired outcome, and “Implementation” to the fidelity with which the designed intervention is carried out. Our primary outcome in Aim 1 is the feasibility of recruitment or reach, i.e., the proportion of eligible, approached patients who enroll in the study. Our primary outcome in Aim 2 is the feasibility of retention, i.e., the proportion of eligible participants who complete 3-month and 6-month follow-up data. We will also assess the feasibility of re-randomization, i.e., the proportion of eligible participants in Stage 1 who are re-randomized to a stage 2 intervention. To assess potential effectiveness, we will utilize the 10-item USDA FSSM [7]. The FSSM was developed using a Rasch model, which supports an interval interpretation of the raw score and can be evaluated as a continuous or binary outcome (food secure vs food insecure) [7]. We will assess FI as a continuous outcome in our primary analysis and as a binary outcome in secondary analyses. We will conduct process evaluation to document intervention delivery and fidelity. We will assess how many participants enrolled in WIC, how often participants met with the WIC provider, how often participants received the Produce Rx, the number of MTM delivered, and adherence to the MTM. The navigator will also maintain a log of their interactions with participants, including the mode of delivery (phone, in-person), time spent, and what was discussed. We plan to evaluate additional secondary or exploratory outcomes as included in Table 2.

### 2.7. Data collection

We will administer surveys at baseline (prior to randomization), 3 months (prior to re-randomization), and 6 months. To minimize participant burden, follow-up assessments will be conducted by phone and participants will be sent the survey by email ahead of the call. Participants will be called up to 5 times at each time point at which measures are collected. Because all participants will be patients at AHWFB, we will also be able to follow-up with participants at their prenatal care visits if we are unable to reach them by phone. We will provide graduated compensation ($20 at baseline, $40 at 3 months, and $60 at 6 months) to reduce the risk of loss to follow up. Surveys will be collected using REDCap, a secure application for building and managing surveys and databases. Participants will complete the surveys on a tablet or computer at their baseline visit and over the phone at follow-up visits. We will also extract data from the EHR regarding participants’ demographics, clinical characteristics, and outcomes (Table 2). Study staff will place CGM devices on participants at the initial in-person baseline visit and mail them to participants at 6 months to self-place. Participants will receive video and written instructions on the CGMs. After 14 days, staff will instruct participants on device removal. The device is painless to apply, requires no interaction after placement, and should not hinder activities of daily life. The device results will be blinded to avoid participants changing behavior based on the readings.

As quantitative measures may not fully capture the complex behavioral and contextual processes, an embedded qualitative approach has been included to produce a richer understanding of the interventions’ effects. We will conduct semi-structured interviews (in English or Spanish) with 2 groups of participants at the end of their 6-month follow-up: participants who 1) had a ≥ 2-point decrease in FI (*n* = 15) and 2) did not have ≥2-point decrease in FI (n = 15). The sample size of 30 participants should be adequate to reach thematic saturation, but we will have flexibility to expand if warranted. Interviews will help generate hypotheses about potential mechanisms of intervention effects, inform future modifications, assess acceptability of the interventions, and help further develop an effective adaptive intervention. Those who complete an interview will receive an additional $25.

### 2.8. Power and sample size

As this is a pilot study, it is primarily designed to determine the feasibility of recruitment, re-randomization, retention, data collection, and adaptively providing FI interventions. Data from this study will provide critical information to refine the study protocol and estimate measures of variance to assist in sample size calculations to inform a future fully-powered SMART. To justify our plan to enroll a target number of study participants in this study, we will follow a confidence interval-based approach [47,48]. Compliance, defined as *p* = 0.9, will be used as the primary outcome in this calculation. We aim to recruit a sufficient number of participants, n, to obtain a margin of error of 0.075 around estimated compliance with 95 % confidence. To conservatively account for up to 25 % attrition, we aim to recruit for a final enrollment of *n* = 60. A sample size of 60 also provides a minimum probability of 80 % of observing at least 5 participants in each subgroup assuming a 40 % response rate for the Stage 1 interventions and 25 % attrition across the entire study period [49,50]. To calculate the power and sample size for a fully-powered trial, both the potential effect size and Stage 1 intervention response rate (proportion of participants who are re-randomized) are necessary for a SMART design. As this is the first study to test an adaptive food insecurity intervention among pregnant individuals, results from this pilot will provide critical information to plan the future trial.

### 2.9. Statistical analysis

In accordance with intention-to-treat, all 60 participants randomized in stage 1 will be included in all analyses. We will summarize participant characteristics at baseline using means and standard deviations (or medians and interquartile ranges) for continuous variables and frequencies and percentages for discrete variables. We will summarize the distribution of FI and secondary outcomes at baseline, 3 months, and 6 months. Our approach to handling incomplete or missing data will depend on the causes and amount. We will determine the likely reasons for missing data (ignorable or not ignorable) and use appropriate methods to address those reasons (e.g., multiple imputation for ignorable). We will use a time-ordered conditional imputation strategy specifically designed for SMART data if we perform multiple imputation [51].

For Aim 1, we will use descriptive statistics to summarize the feasibility (proportion of eligible patients who enrolled). We will also assess the change in FI, dietary intake, food expenditures, psychological outcomes, and healthcare use based on survey responses and EHR data extraction at baseline and 3 months. Given our small sample size, we do not expect to see significant differences in outcomes between participants who were randomized to WIC referral versus those randomized to WIC referral + navigation. Although not the goal or expected, a sample size of 60 does provide sufficient power (0.9) to detect a 2-point difference (SD: 2.0) between the 2 arms with an alpha of 5 % and assuming up to a 25 % loss to follow-up (22 participants per group) using a two-sample *t*-test.

For Aim 2, participants who have a suboptimal improvement in FI (<2-point) at 3 months will be re-randomized in stage 2 resulting in four embedded adaptive interventions (Table 3). To evaluate change in outcomes over time across the four adaptive interventions in the SMART design, piecewise (segmented) marginal mean regression models (fit using generalized estimating equations (GEE)) will be used [52]. In this pilot study, we do not expect to see significant differences between interventions given our small sample size. We will use descriptive statistics and two-stage piecewise GEE regression models to assess differences in outcomes. The fit of this model to our data will inform estimates of variance and the correlation matrix that are necessary to conduct piecewise regression models to assess the differences in a future, fully-powered SMART.

For Aim 3, the semi-structured interviews will occur via telephone and be digitally recorded and transcribed (and translated), with identifying data removed, and guided by a semi-structured interview guide. The guide will be based on the RE-AIM framework. Analyses will be conducted by 2 trained staff, working in parallel, guided by the constant comparison method, and informed by grounded theory methodologies [53–56]. This approach is well suited for systematically uncovering participant experiences and comparing them within and across groups. Analytic steps will include: 1) after each interview, interviewers will document field notes; 2) transcripts and field notes will be entered into Atlas.ti software (Berlin, Germany) for coding and analysis; 3) we will develop a preliminary codebook to include pre-determined deductive codes and inductive codes that emerge from the data; 4) coding differences will be discussed and negotiated until consensus is reached and high inter-rater reliability (k ≥ 0.80) is achieved; 5) similarities and differences will be examined and themes developed; 6) we will “member check” themes to refine and establish the validity of the results. From these rich data, we will gain a greater understanding of how and why interventions produced the outcomes they did and develop a conceptual understanding of how interventions to address FI can most effectively improve care for pregnant patients.

## 3. Discussion

We began recruitment June 2025 and anticipate completing enrollment by the end of 2026. We will begin qualitative data collection after a sufficient number of participants complete the 6-month intervention period (winter 2025/2026). Final results are anticipated in fall 2027. There are several limitations to this study that should be acknowledged. First, this is a pilot study, so the study is not powered to detect differences in the intervention arms or the embedded adaptive interventions. Second, this study will be conducted at obstetric clinics from one health system, so the results may not be generalizable to other settings. Third, although we have taken steps to minimize participant burden, research participation is inherently burdensome, and patients who participate may differ from populations who are pregnant and living with FI.

FI during pregnancy has been associated with numerous negative maternal and infant health outcomes [9–13]. Increasingly, studies are testing Food is Medicine interventions to assist pregnant individuals from food-insecure households as part of routine clinical care [22,23,57]. The interventions tested in these studies have shown promise in reducing FI and improving maternal and infant health outcomes and included referring patients to nutrition support programs, providing Produce Rx, and delivering MTM. However, this study will be the first to develop and test an adaptive intervention to address FI in pregnant patients. An adaptive FI intervention could reduce costs and burden to both participants and clinicians by providing no more treatment than is needed for food-insecure participants who respond to a less intensive approach and redirecting the saved resources to participants who need a more intensive approach. An adaptive FI intervention could also be more efficacious in improving maternal and child health outcomes by providing additional support to patients most in need. Given the growing interest among healthcare systems and insurers in integrating FI interventions in clinical care, an efficacious adaptive intervention could be broadly disseminated across healthcare settings.

## Figures and Tables

**Table 1.** Inclusion and exclusion criteria.

Inclusion	Exclusion
1. ≥18 years of age	1. Planning on moving out of the area within 6 months
2. Confirmed viable pregnancy by their obstetrician or midwife based on urine pregnancy test and ultrasound	2. Severe cognitive impairment or major psychiatric illness that prevents consent or serious medical condition which either limits life expectancy or requires active management (e.g., certain cancers)
3. Experience FI based on the 2-item Hunger Vital Sign^™^	3. Lack safe, stable residence or the ability to store the MTM
4. Speaks English or Spanish	4. Lack of a telephone
5. Not currently enrolled in WIC	5. Severe food allergy or require a specialized diet (e.g., Celiac)
6. First trimester at the time of the initial prenatal visit	

**Table 2.** Study outcomes and measures.

Measure	Potential sample measures and citations	Data source	Month
Primary outcomes			
Feasibility of recruitment/proportion recruited (**Aim 1**)	Proportion of eligible patients who enroll	Study logs	0
Feasibility of retention/proportion retained (**Aim 2**)	Proportion of participants who complete follow-up data collection	Study logs	6
Feasibility of re-randomization/proportion rerandomized (**Aim 2**)	Proportion of eligible participants who are re-randomized to stage 2 interventions	Study logs	3
Food insecurity (**Aim 1 and 2**)	10-item USDA FSSM with 30-day reference period [7]	Survey	0, 3, 6
Maternal outcomes			
Gestational Diabetes Mellitus (GDM)	One-step oral glucose tolerance test shows ≥2 abnormal values consistent with diagnostic criteria for GDM [58], or incident diagnosis of GDM added during pregnancy	EHR data extraction	6
Glucose Homeostasis (Hypo or Hyperglycemia, glucose variability)	14-day monitoring of glucose for episodes of hypo/hyperglycemia and glucose coefficient of variation	CGM	0, 6
Excess Gestational Weight Gain	If pre-pregnancy BMI ≥30, gaining ≥20lbs; if BMI 25–29.9, gaining ≥25lbs; if BMI <25, gaining ≥35lbs [59]	EHR data extraction	Post-delivery
Pre-eclampsia	Incident diagnosis at obstetric, emergency department or hospital encounter	EHR data extraction	Post-delivery
Infant outcomes			
Birth weight	Low birth weight (Weight at birth ≤2500 g (5.5lbs)); Macrosomia (Weight at birth ≥4500 g (9.9lbs) [60]	EHR data extraction	Post-delivery
Preterm Delivery	Delivery prior to 37 weeks gestation [60]	EHR data extraction	Post-delivery
Post delivery	Miscarriage, mode of delivery, APGARS, breastfeeding status	EHR data extraction	Post-delivery
*Nutritional*			
Food expenditures	Out-of-pocket monthly food expenditures [61]	Survey	0, 3, 6
Dietary intake	National Cancer Institute Fruit and Vegetable Screener	Survey	0, 3, 6
Food consumption diaries	Diet adherence (MTM only)	Participant report	Daily
*Psychological*			
Depressive symptoms	Patient Health Questionnaire-9 (PHQ-9) [62,63]	EHR data extraction	0, 3, 6
Stress	Perceived stress scale [64]	Survey	0, 3, 6
*Compensatory*			
Healthcare use	Number of prenatal visits, missed appointments, emergency department use, hospitalization	EHR data extraction	0, 3, 6
*Covariates*			
Demographics	Age, race, ethnicity, sex, insurance status	Survey	0
Clinical characteristics	Pre-existing maternal comorbidities (e.g., Type 2 diabetes), gestational age at first visit, initial body-mass index, smoking status, preferred language	EHR data extraction	0, 6
Health-related social needs	Housing stability, transportation barriers [16]	EHR data extraction	0, 3, 6
Socioeconomic characteristics	Education, marital status, household size, income	Survey	0

**Table 3.** Embedded adaptive interventions.

Intervention	First-stage intervention	Status at end of first-stage	Second-stage intervention
1	WIC referral	Improvement	–
		Suboptimal improvement	Produce Rx
2	WIC referral	Improvement	–
		Suboptimal improvement	MTM
3	WIC referral + navigation	Improvement	–
		Sub-optimal improvement	Produce Rx
4	WIC referral + navigation	Improvement	–
		Sub-optimal improvement	MTM

MTM-medically tailored meals; Produce Rx-produce prescriptions; WIC-Special Supplemental Nutrition Assistance Program for Women, Infants, and Children.

## Data Availability

No data was used for the research described in the article.

## Abbreviations:

CGM: continuous glucose monitor
EHR: electronic health record
FI: food insecurity
FSSM: Food Security Survey Module
GDM: gestational diabetes mellitus
IRB: Institutional Review Board
MTM: medically-tailored meals
SMART: Sequential Multiple Assignment Randomized Trial
SNAP: Supplemental Nutrition Assistance Program
USDA: US Department of Agriculture
WIC: Special Supplemental Nutrition Program for Women, Infants, and Children
